# Correction: Folkertsma et al. The Role of Disease Acceptance and Perceived Control in the Subjective Health Experience Model for Immune-Mediated Inflammatory Diseases. *Biomedicines* 2025, *13*, 538

**DOI:** 10.3390/biomedicines14091917

**Published:** 2026-08-27

**Authors:** Tessa S. Folkertsma, Sjaak Bloem, Robert M. Vodegel, Reinhard Bos, Greetje J. Tack

**Affiliations:** 1 Department of Dermatology, Medisch Centrum Leeuwarden, MCL—Medical Center Leeuwarden, Henri Dunantweg 2, 8934 AD Leeuwarden, The Netherlands; 2 Department of Rheumatology, Medisch Centrum Leeuwarden, MCL—Medical Center Leeuwarden, Henri Dunantweg 2, 8934 AD Leeuwarden, The Netherlands; r.bos@mcl.nl; 3 Department of Gastroenterology, Medisch Centrum Leeuwarden, MCL—Medical Center Leeuwarden, Henri Dunantweg 2, 8934 AD Leeuwarden, The Netherlands; greetje.tack@mcl.nl; 4 Center for Marketing & Supply Chain Management, Nyenrode Business University, 3621 BG Breukelen, The Netherlands; s.bloem@nyenrode.nl

## Title Correction

The title of the original publication [1], “Disease Acceptance and Control from the Subjective Health Experience Model as Health Perception Predictors in Immune-Mediated Inflammatory Diseases”, incorrectly implies that disease acceptance and perceived control predict health perception. The models tested in the study specify the opposite direction, with health perception predicting perceived control and disease acceptance, which subsequently contribute to subjective health experience.

A correction has been made to the title:

“The Role of Disease Acceptance and Perceived Control in the Subjective Health Experience Model for Immune-Mediated Inflammatory Diseases”.

## Error in Figure 2

In the original publication [1], there was a mistake in Figure 2, Model 1 as published. The standardised path coefficients for the relationships from “health perception” to “perceived control” and from “health perception” to “disease acceptance” (β in the arrows leading from the “Health perception” box to the “Control” and “Acceptance” boxes respectively) were inadvertently reported without their negative signs in the path coefficient labels of the corresponding model figure. The corrected Figure 2 appears below. 

## Text Correction

The standardised path β coefficients for the relationships from “health perception” to “perceived control” and from “health perception” to “disease acceptance”, in all four models described in Figure 2, were inadvertently reported without their negative signs in Section 3. Results of the original publication [1].

A correction has been made to Sections 3.3.1–3.3.4, to include the negative signs for the respective β coefficients.

The authors state that the scientific conclusions are unaffected. This correction was approved by the Academic Editor. The original publication has also been updated.

## Figures and Tables

**Figure 2:**
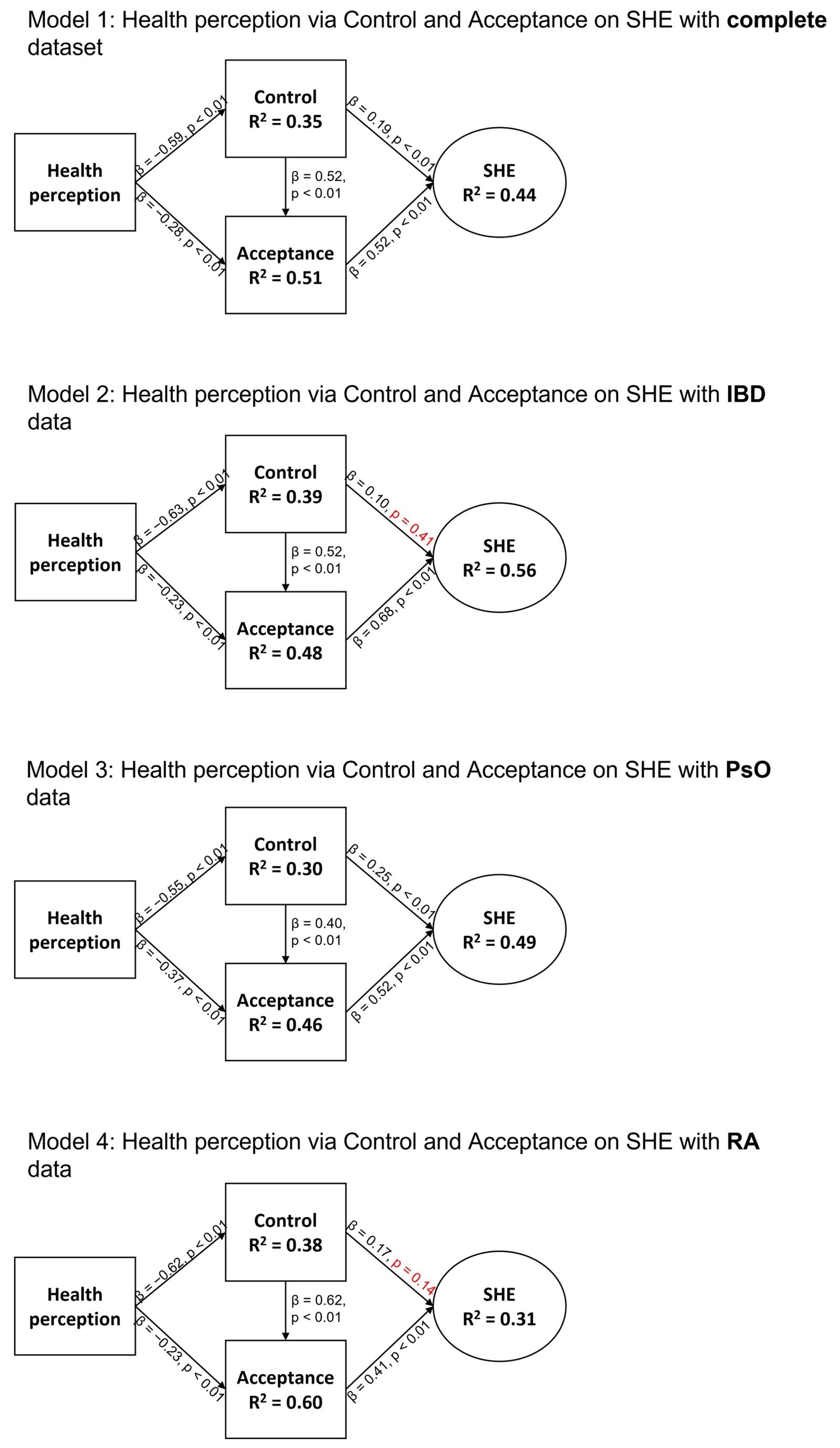
Characteristics of the SHE model for the total study population and for disease-specific data. Health perception was measured via the EQ-5D-5L questionnaire, and acceptance, control, and SHE were measured via the SHE questionnaire. Non-significant *p*-values (*p* > 0.05) are indicated in red.
